# Target response controlled enzyme activity switch for multimodal biosensing detection

**DOI:** 10.1186/s12951-023-01860-z

**Published:** 2023-04-08

**Authors:** Lu Zhang, Haiping Wu, Yirong Chen, Songzhi Zhang, Mingxuan Song, Changjin Liu, Jia Li, Wei Cheng, Shijia Ding

**Affiliations:** 1 Key Laboratory of Clinical Laboratory Diagnostics (Ministry of Education), College of Laboratory Medicine, Chongqing Medical University, Chongqing, 400016 People’s Republic of China; 2 Department of Laboratory Medicine, The Fifth People’s Hospital of Chongqing, Chongqing, 400062 China; 3 The Center for Clinical Molecular Medical Detection, The First Affiliated Hospital of Chongqing Medical University, Chongqing, 400016 People’s Republic of China

**Keywords:** Hemin, Controlled enzyme activity, Dimerization inactivation, G4-Hemin DNAzyme, Multimodal detection

## Abstract

How to achieve delicate regulation of enzyme activity and empower it with more roles is the peak in the field of enzyme catalysis research. Traditional proteases or novel nano-enzymes are unable to achieve stimulus-responsive activity modulation due to their own structural limitations. Here, we propose a novel Controllable Enzyme Activity Switch, CEAS, based on hemin aggregation regulation, to deeply explore its regulatory mechanism and develop multimodal biosensing applications. The core of CEAS relies on the dimerizable inactivation of catalytically active center hemin and utilizes a DNA template to orderly guide the G4-Hemin DNAzyme to tightly bind to DNA-Hemin, thereby shutting down the catalytic ability. By customizing the design of the guide template, different target stimulus responses lead to hemin dimerization dissociation and restore the synergistic catalysis of G4-Hemin and DNA-Hemin, thus achieving a target-regulated enzymatic activity switch. Moreover, the programmability of CEAS allowed it easy to couple with a variety of DNA recognition and amplification techniques, thus developing a series of visual protein detection systems and highly sensitive fluorescent detection systems with excellent bioanalytical performance. Therefore, the construction of CEAS is expected to break the limitation of conventional enzymes that cannot be targetable regulated, thus enabling customizable enzymatic reaction systems and providing a new paradigm for controllable enzyme activities.

**Graphical Abstract:**

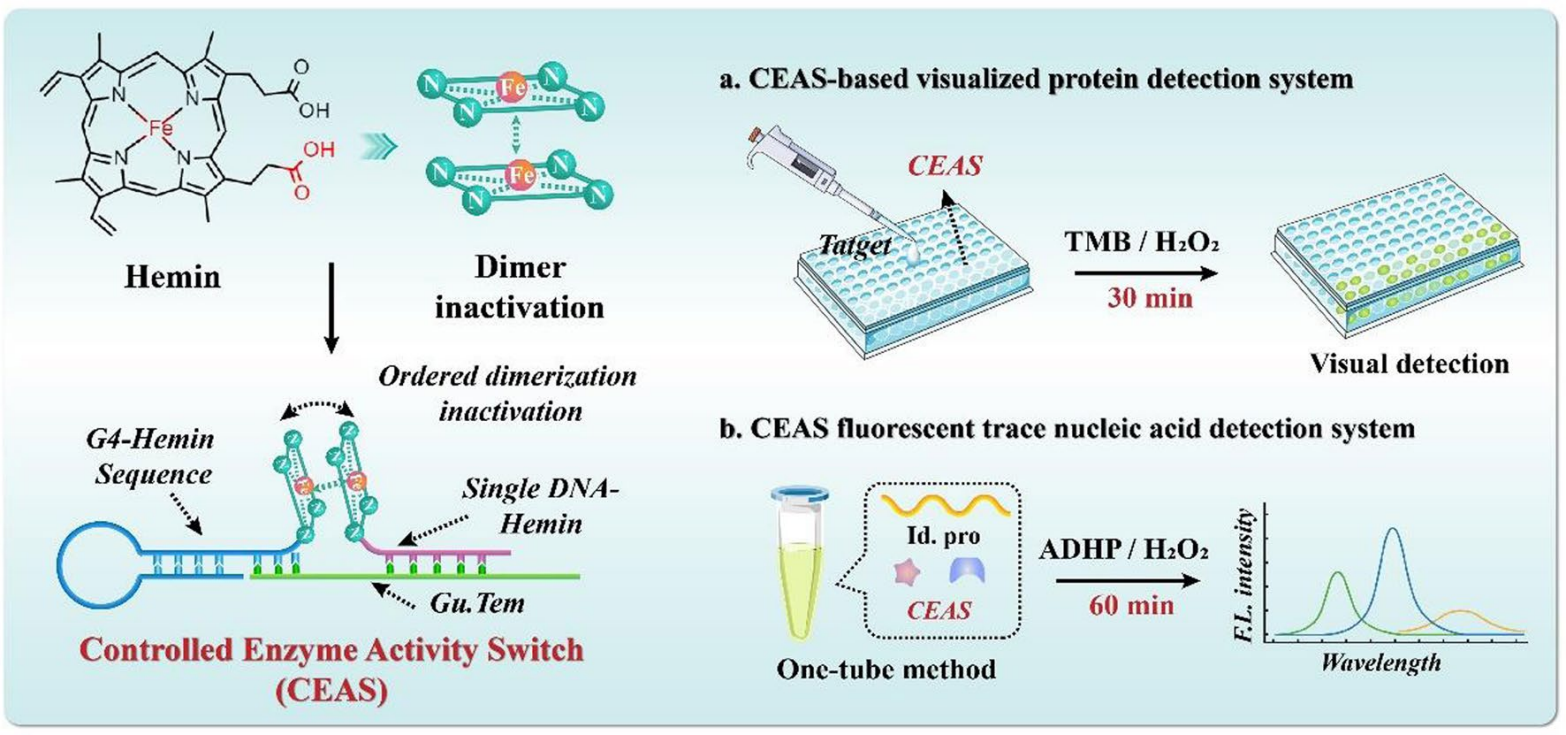

**Supplementary Information:**

The online version contains supplementary material available at 10.1186/s12951-023-01860-z.

## Introduction

Enzyme-catalyzed biosensing technologies, represented by enzyme-linked immunosorbent assays (ELISA) and others, have been widely applied in the detection of disease biomarkers [1–4]. Protease tags, such as horseradish peroxidase (HRP) [5] and lactate dehydrogenase [6], exert efficient and specific substrate catalytic properties to achieve highly sensitive signal output through fluorescent and colorimetric platforms [7]. However, due to the uncontrolled catalytic activity of protease tag, ELISA technique and others requires coupling of tedious sample identification and background removal steps. In addition, the inactivation and difficulty in labeling of protease creates a harsh operating environment and low sensitivity. Therefore, in the era of increasingly efficient precision medicine, traditional enzymatic biosensing systems are struggling to meet clinical requirements, and there is an urgent demand for assay systems with higher analytical performance for clinical applications. In order to abandon the harsh environment of protease bio-applications and the high susceptibility to deactivation, various novel nanomimetic enzymes with catalytic functions have been created by the intrinsic physicochemical properties of nanoparticles [8–11]. For example, classical Fe_3_O_4_ nanoparticles can highly mimic peroxidase activity [12]; small size gold nanoparticles can also catalyze glucose to perform the biological properties of glucose oxidase [13, 14]. These nanoenzymes gradually rival the performance of natural enzymes in terms of catalytic activity and substrate specificity, greatly broadening the application scenario of enzyme-catalyzed sensing strategies [15]. However, in both protease and nanoenzyme-based biosensing detection strategies, the enzyme is positioned only as a “fixed” catalytic machine that functions as a signal output [16, 17]. The introduction of enzyme often directly triggers the output of substrate signal, without achieving controlled activity regulation. This uncontrollable enzyme signal output pattern determines the requirement of coupling complicated signal specificity identification, screening and other pre-paving, while itself only as the final finale. Therefore, at a time of biosensing technology moving toward more intelligence and refinement, how to endow enzymes with more abundant functions and achieve simpler and more accurate activity regulation is a key issue worthy of in-depth exploration.

Precise base complementary pairing and adjustable multidimensional structure enable DNA, the star molecule, to be labeled with the advantages of programmability, refinability, and multi-biological functionality [18–21]. Through specific base design, DNA sequences can be folded to form high-dimensional spatial structures that mimic the natural enzyme catalytic spatial conformation and combine with cofactors to exert efficient enzyme-like catalytic properties [22–25]. This category of nucleic acid mimetic enzymes, represented by G4-Hemin DNAzyme, possesses irreplaceable biological properties compared to conventional inorganic nanosized enzymes, inheriting the advantages of programmability and tunability of DNA molecules. G4-Hemin DNAzyme with peroxidase-like activity has been widely employed in various field such as biosensing and molecular therapeutics, exhibiting unparalleled application potential [26–28]. However, the classical G4-Hemin DNAzyme is limited by its lower catalytic activity and an equally uncontrollable regulation of activity. In-depth dissection of the catalytic conformation of G4-Hemin reveals that the hemin monomer as the catalytic active center folds with the G4 functional sequence to form a spatial structure through π–π stacking, creating a similar construct to that of HRP [29]. Hemin monomer molecules exhibit low water solubility and tend to deactivate by dimerization, which may be one of the reasons for the low catalytic performance of conventional G4-Hemin DNAzyme [30, 31]. On the other hand, this phenomenon also suggests that the activity of G4-Hemin DNAzyme may be effectively controlled by the aggregation morphology of hemin itself, which provides an ingenious idea for the development of effective controllable enzyme activity.

**Scheme 1:**
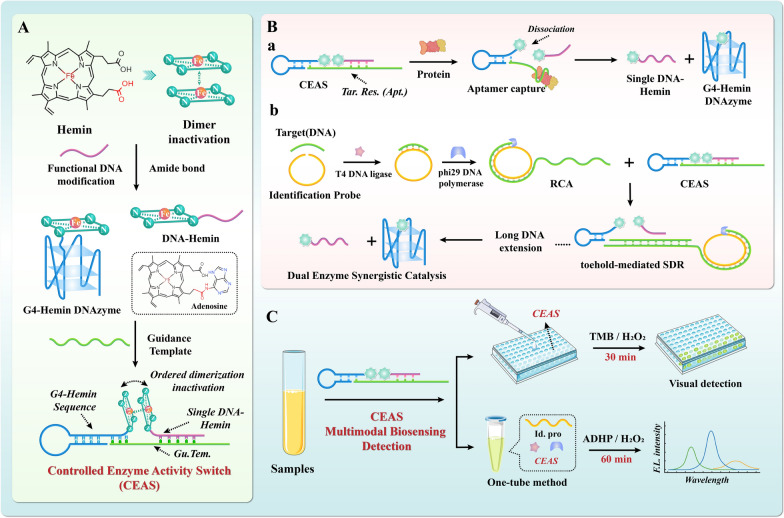
Schematic diagram of the construction of CEAS and its application to multimodal detection. **A** Construction of the Controlled Enzyme Activity Switch (CEAS). **B** Schematic representation of **a** CEAS system respond to protein targets and deconstruct into active catalytic modes. **b** CEAS coupled with RCA, SDR is sensitive and rapid for nucleic acid target detection. **C** Rapid and efficient application of CEAS multimodal biosensing detection in practice. *Gu. Tem.* guidance template, *Tar. Res. (Apt.)* target response (aptamer), *Id.pro* identification probe

In summary, we propose to construct a Controllable Enzyme Activity Switch (CEAS) to achieve target-responsive enzyme catalytic modulation for purposeful signal output, with multimodal detection application potential. As shown in Scheme 1, modification of hemin monomers with functional nucleic acids directs the orderly aggregation of hemin monomers through the DNA guidance template (Gu.Tem.), which allows bursting of G4-Hemin DNAzyme activity in the absence of the target. In contrast, when different types of targets are present, aptamer recognition for target response (Tar. Res. (Apt.)) or toehold-mediated strand displacement reaction (SDR) enables dimer dissociation of DNA-associated hemin monomers through the un-stranding process of DNA hybridization. The G4 functional sequence forms a high-performance peroxidase-like enzyme with coupled hemin and constitutes a synergistic catalytic system with the free DNA-Hemin adduct, thus significantly enhancing the analytical performance. Relying on different choices of targets and catalytic substrates, the CEAS is suitable for multi-modal assay applications and can be easily coupled with nucleic acid amplification strategies, demonstrating the performance advantages of high sensitivity, high specificity, simplicity, precision and controllability, promising a revolutionary alternative to enzyme-catalyzed clinical assays.

## Experimental

### Reagents and materials

The sequence of DNA oligonucleotides used in this work and the related abbreviations are listed in Additional file 1: Table S1. Unmodified DNA oligonucleotides were synthesized by Sangon Biotech (Shanghai) Co. Ltd. Hemin-modified, HPLC-purified oligonucleotides were obtained from Takara Biotechnology (Dalian, China). Their concentrations were determined by UV absorbance at 260 nm using the molar extinction coefficients provided by the Oligo-Analyzer v3.1 tool (http://sg.idtdna.com/calc/analyzer). All oligonucleotides were dissolved in Tris-ethylene diamine tetra-acetic acid buffer (pH 8.0, 10 mM Tris–HCl, 1 mM EDTA) and stored at − 20 °C. All other reagents were of analytical grade. All buffer solutions were prepared using Millipore-Q water (≥ 18 M, Milli-Q, Millipore). Hemin was dissolved in dimethyl sulfoxide as a stock solution then diluted to the required concentration by 50 mM Tris–HCl buffer (pH 7.0, 25 mM KCl). Freshly prepared TMB was dissolved in ultrapure water to a concentration of 4 mM. Human Thrombin ELISA KIT was purchased from CUSABIO Biotech. All the chemicals were obtained from Sigma. In addition, the fluorescent substrate ADHP (OxiRed probe) was dissolved in Tris–HCl buffer (pH 10.0) at a concentration of 10 M prior to use.

### Apparatus

The Cary Eclipse fluorescence spectrophotometer (Agilent Technologies, Palo Alto, CA) was employed to measure all the fluorescence spectra. The nucleic acid concentration was quantified by the NanoDrop 1000 spectrophotometer (Thermo Scientific, USA). Ultraviolet–visible (UV–vis) absorption spectra were recorded with a UV-2550 spectrophotometer (Shimadzu, Japan). The polyacrylamide gel electrophoresis (PAGE) was performed on a Bio-Rad electrophoresis analyzer (Bio-Rad, USA) and imaged on Bio-Rad ChemDoc XRS (Bio-Rad, USA).

### Assembly of CEAS

According to the principle of complementary base pairing, 10 μM DNA-Hemin strand (A strand), G4-Hemin strands (B strand) and guidance template (C strand) were simultaneously mixed in 100 μL TA-Mg^2+^ buffer, annealed at 95 °C for 5 min to form complete CEAS, then cooled to room temperature and stored at 4 °C before use. In addition, there was another assembly mode option, which was carried out at 37 °C and was specifically described as follows: the B strand at 95 °C for 5 min to form a complete hairpin structure, and then incubated with the A strand and C strand at 37 °C for 1 h. Finally, the CEAS was separated and stored at 4 °C. Exploring the proportion of strands is necessary for the catalytic performance of CEAS during their construction. The B and C strands of 1 μM were added to binding buffer in proportion to the A strands of 0.5, 1, 1.25, 1.5, and 2, respectively, and then annealed at 95 °C for 5 min to room temperature. In addition, in order to achieve the best blocking effect of the CEAS, the number of hemin interval bases was optimized. The detailed assembly steps were as follows: 1 μM G4-Hemin strands (B strand), guidance template (C strand) and DNA-Hemin (A strands) were distributed with − 2, − 1, 0, 1, 2 T bases spaced and then annealed at 95 °C for 5 min in 15 mM TA-Mg^2+^ buffer. The signal-to-blank ratio of each group was verified by the fluorescent signal system and then the optimal reaction conditions were selected among them.

### Catalytic performance of CEAS

Briefly, 10 μL of CEAS (100 nM) was incubated in 50 mM Tris–HCl buffer for 1 h at 37 °C. When the reaction was carried out, the B strand was completely folded into the G4-Hemin complex. After subsequent addition of TMB (4 mM) and H_2_O_2_ (50 mM), the folded formed G4-Hemin complex catalyzed substrate, producing products that could be analyzed by UV–visible spectroscopy in the range of 300–600 nm. Catalytic kinetics experiments were performed on an Agilent Cary100 spectrophotometer at 650 nm for 600 s. At the same time, the ADHP (35 mM) and H_2_O_2_ (50 mM) were added into another group, which could be speculated by the Cary Eclipse fluorescence spectrophotometer at 585 nm (Agilent Technologies, Palo Alto, CA).

### CEAS-based visualization assay system for protein biomarker detection

Thrombin was selected as the model target for visualization system validation. The detailed steps were as follows: thrombin (100 nM) was added to a 100 μL reaction mixture containing 100 nM of CEAS, and incubated at 37 °C for 30 min. And then, 100 μL of 4 mM TMB and 1 μL of 10 M freshly prepared H_2_O_2_ were added and the absorption spectra were recorded in the wavelength range of 300–600 nm after 1 min of reaction at room temperature.

### CEAS-based highly sensitive assay system for trace nucleic acid detection

The highly sensitive CEAS fluorescent trace nucleic acid detection system was verified by using trace HPV DNA as the detection model. Detailed detection steps were shown as follows: 1 μM padlock probe and 1 μM HPV were mixed in T4 DNA ligase buffer (50 mM Tris–HCl, 10 mM MgCl_2_, and 1 mM ATP, pH 7.4). In order to guarantee that padlock probe could fully hybridize with HPV gene, the mixture was heated to 95 °C and slowly cooled down to room temperature (RT). Then, after 30 Weiss U of T4 DNA ligase was added, ligation process was performed for 30 min at 22 °C to form a circular template by the ligation of the 5′-phosphate and 3′-hydroxyl ends of the padlock probe. The ligation reaction was terminated by inactivation at 65 °C for 10 min. The RCA reaction with 25 μL phi29 DNA polymerase reaction buffer (33 mM Tris–acetate, 10 mM Mg-acetate, 66 mM K-acetate, and 0.1% Tween 20, 1 mM DTT) containing prepared circular template, 5 U phi29 DNA polymerase and 2.4 μM dNTP was performed and then 100 nM of CEAS was added simultaneously incubated 37 °C for 30 min. Finally, the above reaction mixture was added in 2.5 μL 35 mM ADHP and 1 μL 3% H_2_O_2_, and the fluorescence signal at 558 nm was measured by the Cary Eclipse fluorescence spectrophotometer (Agilent Technologies, Palo Alto, CA) in the wave length range from 570 to 700 nm.

## Results and discussion

### The assembly of CEAS

**Fig. 1:**
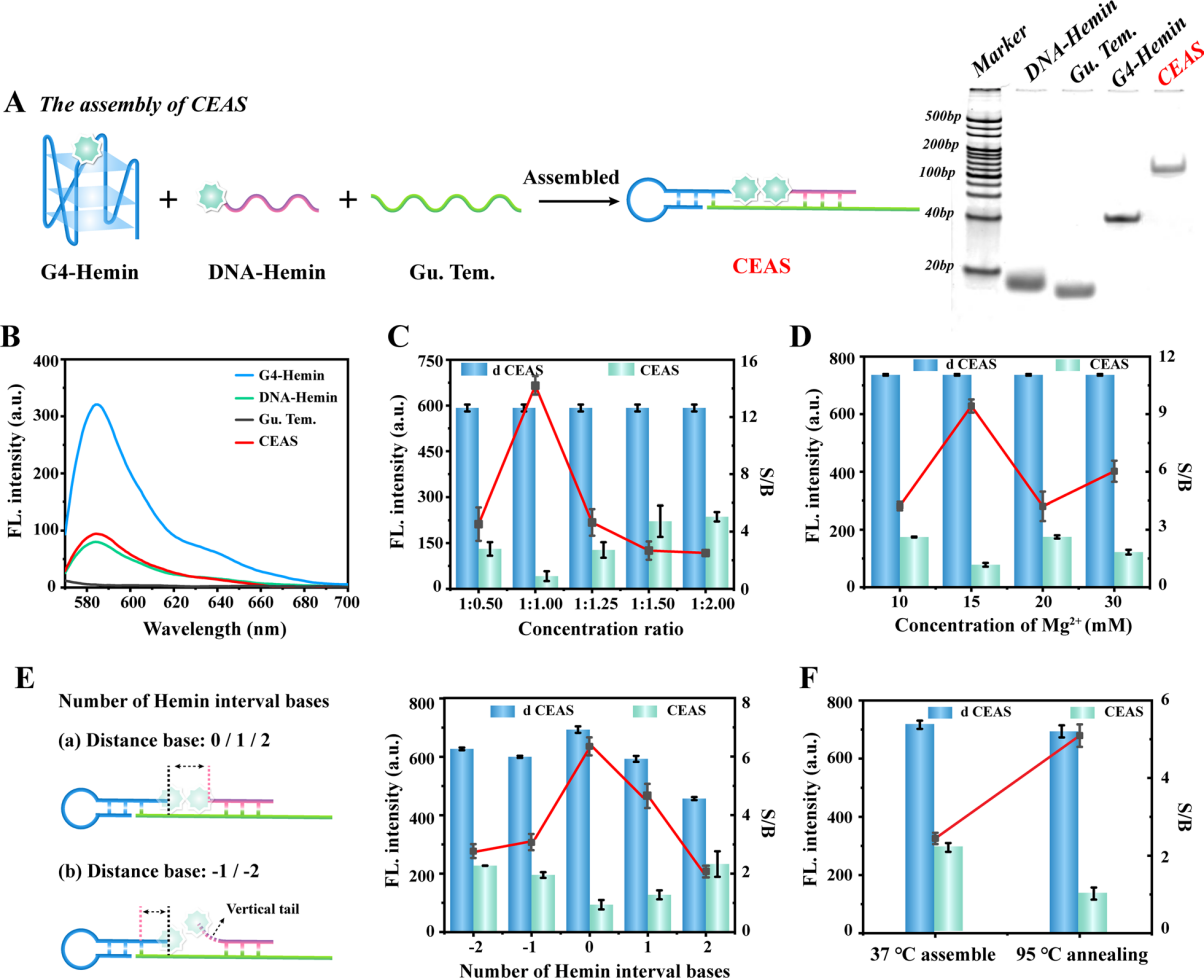
**A** Assembly process of CEAS and verification by PAGE electrophoresis. **B** Fluorescence spectroscopy validation of CEAS assembly. **C** Optimization of concentration ratio of CEAS assembly components. **D** Optimization of Mg^2+^ concentration in the assembly buffer. **E** Optimization of the number of hemin spacer bases. **F** Optimization of CEAS assembly method. Error bars represent standard deviations of three independent experiments. The right Y-axis S/B represents the ratio of signal to blank

As shown in Fig. 1A, CEAS consists of a hairpin structured G4-Hemin DNAzyme, single-stranded DNA-Hemin and a guide template (Gu. Tem.) The DNA template guides the G4-Hemin and DNA-Hemin to hybridize closely, allowing the two hemins to be ordered in close proximity, forming a dimeric inactivation, thus leaving the CEAS catalytic activity in the “OFF” state. Then, the construction of CEAS was characterized by various methods. First, the PAGE electrophoresis map clearly demonstrated the presence of bright bands with larger molecular weight in the last lane and a slower migration rate compared to the other single strand components, indicating that the three single strand components could assemble to form a composite structure (Fig. 1A). Subsequently, the fluorescence spectra of the assembled CEAS were verified. The G4-Hemin DNAzyme could catalyze the strong fluorescent signal of ADHP [32] due to its efficient activity, and the DNA-Hemin monomer also possessed a weak catalytic ability. In contrast, once G4-Hemin and DNA-Hemin formed a CEAS structure via DNA-guided orderly aggregation, the fluorescence signal intensity decreased substantially compared to that of G4-Hemin alone (Fig. 1B). The above results fully indicated the effective assembly of CEAS structure and verified that DNA-guided hemin aggregation could achieve the inhibition of G4-Hemin DNAzyme activity.

In order to better achieve CEAS assembly and reduce the catalytic background signal, a series of essential parameters were fully optimized. The concentration ratio between the three components of G4-Hemin, DNA-Hemin and guide template has a crucial influence on the construction of CEAS. The freeing of either G4-Hemin or DNA-Hemin may cause incomplete burst of catalytic activity, resulting in a strong background signal. Therefore, we assumed a consistent concentration between the guide template and G4-Hemin, and adjusted the concentration ratio of DNA-Hemin for further optimization. The results of Fig. 1C revealed that the optimal signal-to-blank (S/B) ratio could be obtained when the concentration ratio was chosen as 1:1, where d-CEAS indicated the deconstructed CEAS by toehold-mediated SDR and CEAS represented the complete DNAzyme inhibition structure. Therefore, the concentration ratio of 1:1 was taken for the subsequent assembly of CAES. Ion concentration has been also considered as an essential factor affecting DNA assembly [33]. Thus, Mg^2+^ concentration in the assembly buffer was set in a gradient, and the CEAS catalytic signal under different ion conditions was analyzed using the fluorescence system. As shown in Fig. 1D, the S/B ratio reached the peak at the Mg^2+^ concentration of 15 mM, which was regarded as the optimal ion condition. Furthermore, the core of CEAS activity regulation lies in the inactivation of G4-Hemin DNAzyme by DNA-guided hemin aggregation. The base distance between G4-Hemin and DNA-Hemin has the potential to impact on the hemin dimerization. Therefore, the spacing between G4-Hemin and DNA-Hemin was adjusted by inserting different A bases in DNA-Hemin chain. As shown in Fig. 1E, the background signal reached a minimum when no bases were spaced between G4-Hemin and DNA-Hemin, which indicated that tight binding between hemin was achieved. Finally, different CEAS assembly modes were further verified, and the results showed that the co-annealing at 95 °C 5 min was more adequate for hybridization compared with mild condition at 37 °C 1 h, which exhibited lower background signal of free hemin in CEAS (Fig. 1F). The above results fully explored the essential conditions affecting the CEAS assembly and obtained the optimal catalytic S/B ratio.

### The catalytic activity of CEAS

**Fig. 2:**
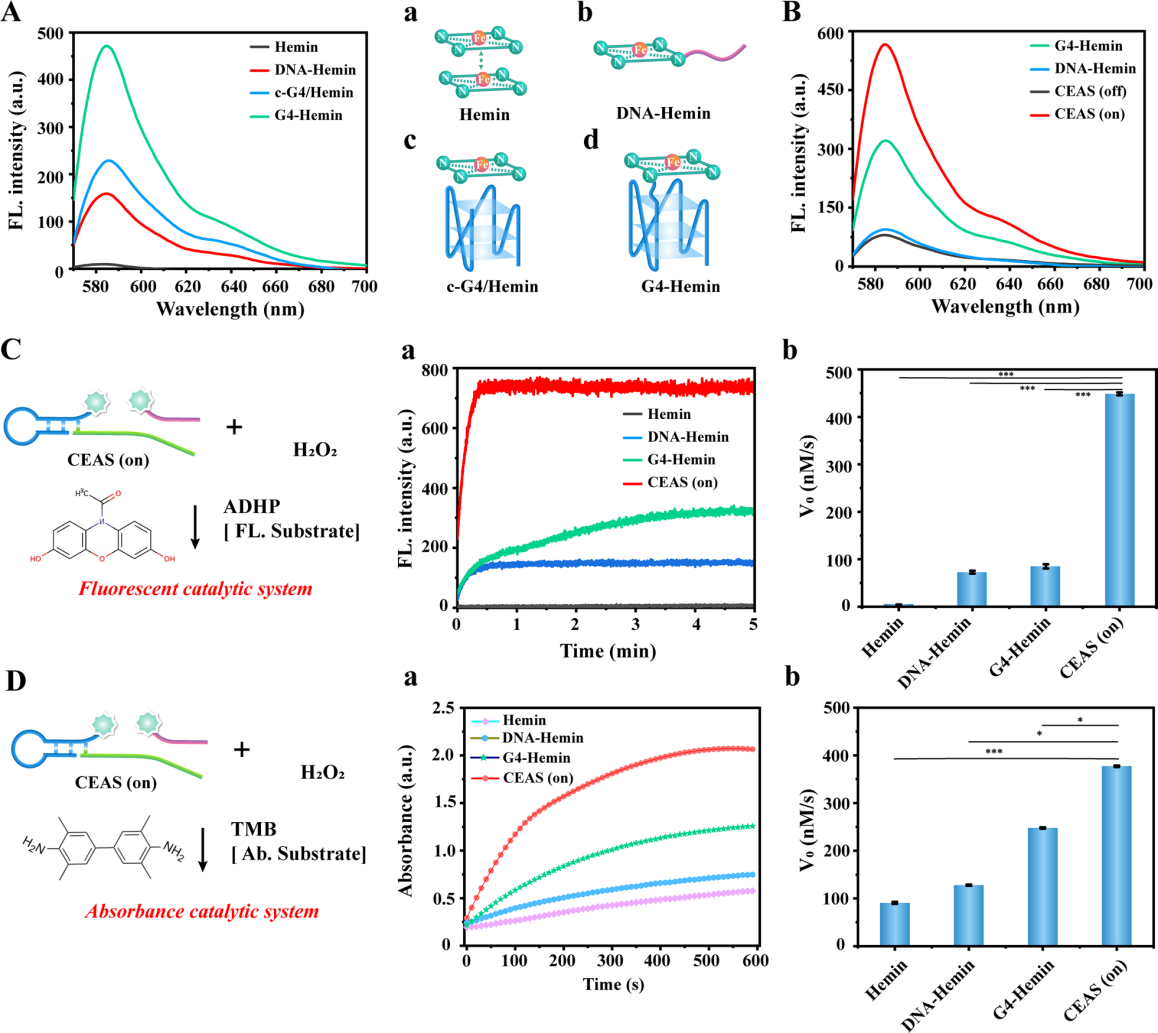
**A** Validation of the catalytic properties of four different hemin model: **a** Hemin monomer; **b** DNA-Hemin; **c** c-G4/Hemin; **d** G4-Hemin. **B** Commissioning verification of CEAS switch states. **C** Catalytic kinetics and initial rate comparison of CEAS fluorescent system. **a**, **b** Represent the kinetics and corresponding initiation rates of the catalytic reaction of Hemin, DNA-Hemin, G4-Hemin, and CEAS to fluorescent substrate ADHP, respectively. **D** Catalytic kinetics and initial rate comparison of CEAS colorimetric systems. **a** Catalytic kinetics and **b** catalytic rate comparison of the CEAS absorbance catalytic system. Error bars represent standard deviations of three independent experiments. *P < 0.05, ***P < 0.001

The main mechanism of CEAS relies on the alteration of the dimerization catalytic properties of hemin, the catalytic activity center of DNAzyme, to achieve enzyme activity modulation. The effect of hemin aggregation state on the catalytic performance of G4-Hemin DNAzyme deserves to be explored first. Figure 2A demonstrated the different forms of hemin as a mimetic enzyme catalyst: (a) Hemin monomer tended to dimerize and deactivate due to its insolubility in water, showing a negligible faint signal in the fluorescence spectrum; (b) Hemin was splice coupled with hydrophilic DNA (Additional file 1: Fig. S1 indicated the results of DNA-Hemin mass spectrometry validation). Owing to the significant increase in water solubility, the dimerization of DNA-Hemin was obviously reduced, and thus the catalytic performance was considerable; (c) the conventional G4/Hemin (c-G4/Hemin) mimetic enzyme possessed a complete catalytic conformation and therefore could effectively catalyze ADHP to produce a significant fluorescent signal output, but the limitation of catalytic performance caused by free hemin aggregation was still unavoidable; (d) the DNA-modified hemin-assembled G4-Hemin DNAzyme exhibited superior catalytic performance compared with the conventional G4-Hemin DNAzyme due to the avoidance of free hemin dimerization. The above results fully demonstrate that the aggregation state of hemin is a key factor affecting the catalytic performance of G4-Hemin DNAzyme, and also confirm the feasibility of achieving controlled enzyme activity switching by regulating hemin aggregation.

Subsequently, the controllable activity of CEAS was tuned under optimal assembly conditions. The fluorescence results showed that the CEAS (off) state was essentially devoid of catalytic activity, while the deconjugated CEAS (on) state could generate a significant fluorescence signal and was stronger compared to DNA-Hemin and G4-Hemin alone (Fig. 2B), which indicated that the free DNA-Hemin and G4-Hemin in the activated CEAS possessed synergistic catalytic effects and contributed to achieve considerable signal enhancement.

Finally, to further explore the catalytic application scenarios of CEAS, its catalytic performance in terms of fluorescence and colorimetric signal output was verified with ADHP/H_2_O_2_ and TMB/H_2_O_2_ systems, respectively. As shown in Fig. 2C, CEAS could catalyze ADHP to reach the signal plateau within 1 min after the activation of deconjugation. The results of corresponding catalytic initial rate (V_0_, nM/s) indicated that the activated CEAS (448 nM/s) increased approximately 5.1-fold compared to G4-Hemin alone (87 nM/s). The catalytic kinetic results in the TMB/H_2_O_2_ system exhibited similar trends to the ADHP/H_2_O_2_ system, both suggesting a higher catalytic performance of the activated CEAS (Fig. 2D). These results indicated that CEAS possessed a wide range of catalytic scenarios and exhibited promising catalytic performance due to the synergistic catalytic effect of G4-Hemin DNAzyme and DNA-Hemin, which was expected to enable easy and highly sensitive detection applications.

### CEAS-based visualization assay system for protein biomarker detection

Based on the above results, CEAS possesses efficient catalytic performance in fluorescence and visual colorimetric assays. Therefore, we constructed a multimodal disease biomarker detection system based on CEAS to validate the potential for bioanalytical applications.

**Fig. 3:**
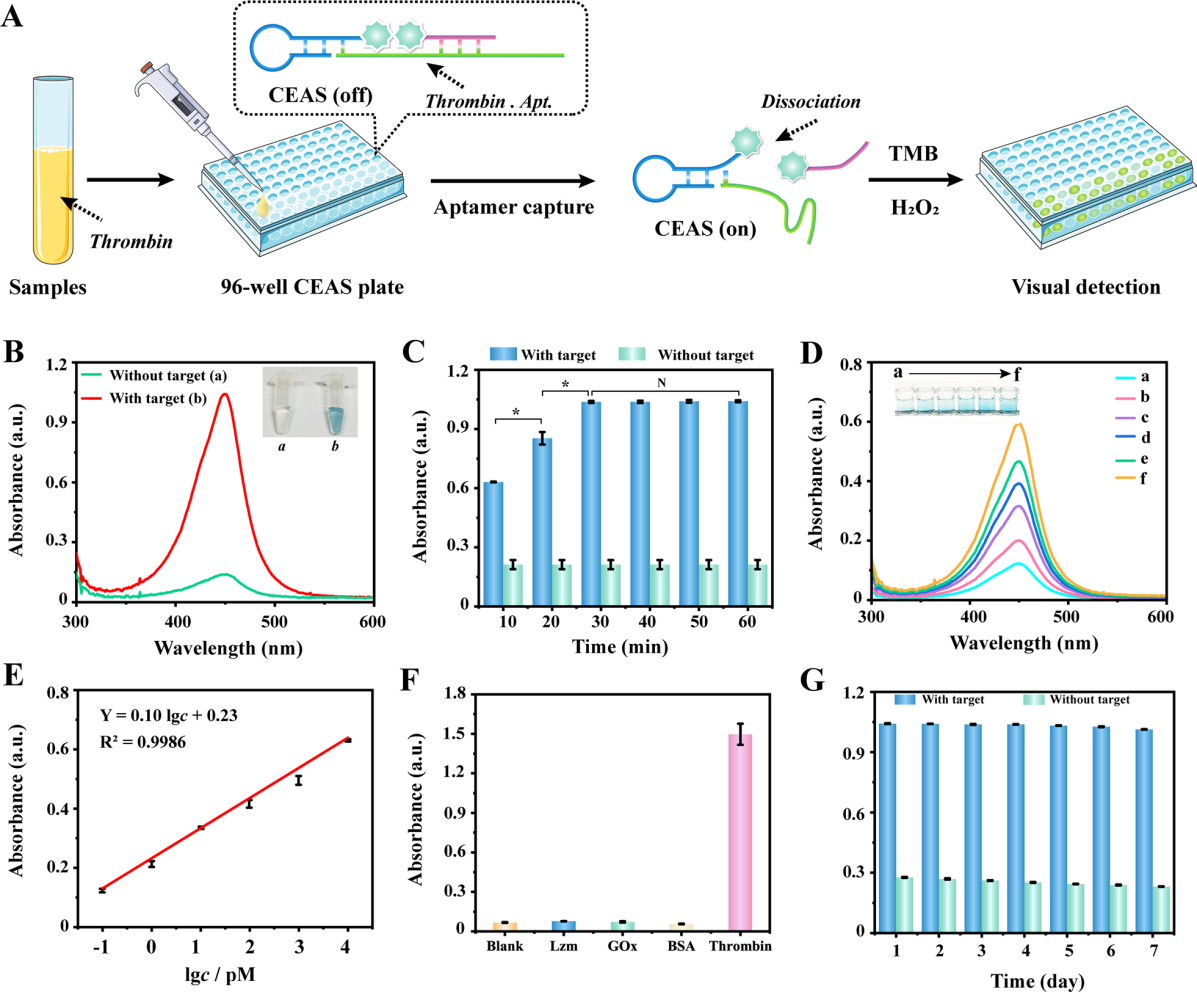
**A** Schematic diagram of the CEAS visualization protein detection system. **B** Feasibility validation of the CEAS visualization assay system. **C** Incubation time optimization of the CEAS visualization assay system. **D** Colorimetric signal responding to **a** 100 fM, **b** 1 pM, **c** 10 pM, **d** 100 pM, **e** 1 nm to **f** 10 nM of thrombin (from **a** to **f**). **E** Linear correlation between the colorimetric intensity and the logarithm of target concentrations ranging from 10^–1^ to 10^4^ pM. **F** Specificity validation of CEAS visualization assay. **G** Stability validation of CEAS visualization assay. Error bars represent standard deviations of three independent experiments. *P < 0.05, *N* no statistical difference

Thrombin was selected as the detection model, and a visualized protein biomarker rapid detection system was proposed to be constructed using the highly programmable CEAS combined with aptamer target recognition technology. As illustrated in Fig. 3A, the guide template in the CEAS assembly system was designed as a thrombin target aptamer. When thrombin existed, it could be specifically recognized by the guide template and caused CEAS to deconjugate. Hemin dimerization was broken in the deconjugation-activated CEAS, releasing G4-Hemin DNAzyme and DNA-Hemin, which could be rapidly visualized for colorimetric detection by synergistically catalyzing TMB and H_2_O_2_.

First, the feasibility of the CEAS-based visualized protein detection system was verified. As depicted in Fig. 3B, the addition of thrombin could be clearly observed as a dark blue color change, while the un-activated CEAS was a transparent solution. The contrasting visualization results fully illustrated the successful construction of the CEAS protein detection system. Subsequently, the reaction time of the visualization assay system was further optimized. The results in Fig. 3C indicated that the colorimetric signal intensity leveled off after 30 min. Compared with conventional immunoassay techniques [34, 35], which require 1–2 h reaction time, the CEAS visualization system greatly shortens the detection time due to the one-step system activation and efficient co-enzyme catalytic performance. The analytical performance of this strategy was further investigated. The color shades of the visualization system corresponded to the concentration gradient of thrombin series. The colorimetric signal exhibited a favorable linear relationship with thrombin concentration in the range of 100 fM–10 nM, and the fitted linear equation was Y = 0.10 × (lg *c*) + 0.23 (R^2^ = 0.9986), where Y is the absorbance and *c* is the concentration of thrombin (Fig. 3D, E). The limit of detection was calculated to reach 1.8 pM according to the 3σ method [36], which meets the demand for quantitative clinical prothrombin assays.

In addition, the targeted identification of aptamer guarantees the specificity of the CEAS visualization assay system. The results of Fig. 3F confirmed that the presence of several different protein interferers, such as Lzm (lysozyme), GOx (Glucose oxidase) and BSA (Bovine serum albumin), did not produce significant signal changes, and only the addition of thrombin showed a remarkable signal increase. Long time signal detection stability was also verified, with signal RSD (Relative standard deviation) controlled within 5% over 7 days (Fig. 3G). Finally, to explore the potential application of this assay system in clinical samples, the CEAS visual protein detection system was used for thrombin detection in serum complex matrices. The spike-recovery of the system was verified to be 99.3–103.20% with an RSD of 1.29–2.45% (Additional file 1: Table S2). The above results demonstrated the superiority of CEAS applied to visualize protein detection with the advantages of rapidity, sensitivity and simplicity, which were attributed to the ingenious design of target directly activating enzyme activity and the efficient enzyme catalytic performance.

### CEAS-based highly sensitive assay system for trace nucleic acid detection

**Fig. 4:**
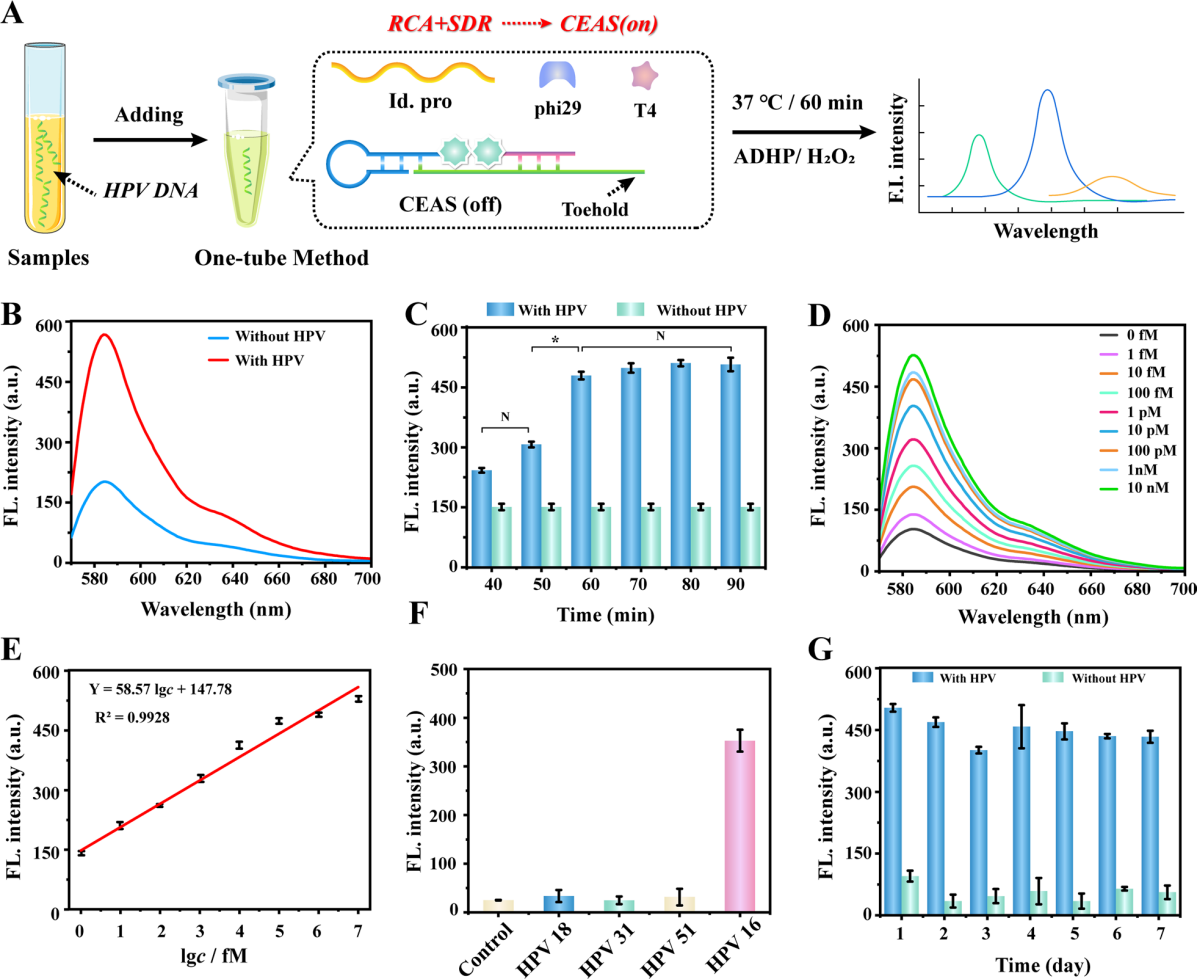
**A** Schematic diagram of the CEAS highly sensitive fluorescence detection system. **B** Feasibility validation of the CEAS highly sensitive fluorescence detection system. **C** Incubation time optimization of the CEAS highly sensitive fluorescence detection system. **D** Fluorescent signal responding to 1 fM, 10 fM, 100 fM, 1 pM, 10 pM, 100 pM, 1 nM and 10 nM of HPV16. **E** Linear correlation between the colorimetric intensity and the logarithm of target concentrations ranging from 10^0^ to 10^7^ fM. **F** Specificity validation of the CEAS highly sensitive fluorescence detection system. **G** Stability validation of the CEAS highly sensitive fluorescence detection system. Error bars represent standard deviations of three independent experiments. *P < 0.05, *N* no statistical difference

To further expand the application scenario of CEAS, a highly sensitive CEAS fluorescent trace nucleic acid detection system was proposed by integrating rolling circle amplification (RCA) and toehold-mediated stand replacement reaction (SDR). This system chose HPV16 (Human Papilloma Virus) DNA as the detection model. In the presence of target, cyclization of the padlock identification probe in the RCA system can be mediated first to form an amplification template. Then, the nucleic acid target serves as the primer for cyclized template to achieve efficient extension by polymerase, producing numerous long single-stranded products with repetitive sequences. The single-stranded products allow for Hemin dimer release by opening the structure of CEAS probe with the toehold-mediated SDR, thus restoring G4-Hemin DNAzyme catalytic activity and achieving highly sensitive signal output in the presence of fluorescent substrate (Fig. 4A).

The feasibility of this fluorescence system was firstly verified. The PAGE electrophoresis results of Additional file 1: Fig. S2 showed a large number of aggregated products in lane 4, indicating that the RCA reaction could be successfully performed. The fluorescence results indicated that a strong fluorescence signal could be generated in the presence of HPV only and an appreciable S/B ratio was obtained (Fig. 4B). Due to the combination of multiple processes, including RCA, SDR and CEAS, the incubation time of the system causes a significant impact on the detection signal. By optimizing a series of time gradients, it could be clearly observed that the signal peak was reached after 60 min (Fig. 4C). The whole process can be briefly summarized as follows two key steps: Preparation of cyclization template: the primers and padlock probe were fully cyclized at 22 °C for 30 min and the mixture of CEAS and RCA amplification (such as dNTP, phi29 DNA polymerase, etc.) was added and amplified at 37 °C for 30 min. Long RCA product obtained allows for CEAS deconstruction with the aid of the toehold, and the RCA process and SDR process can be completed efficiently within 30 min at the same time. This was attributed to the extremely high efficiency of RCA amplification and the high catalytic performance of CEAS. The concentration of ADHP and pH in the fluorescent catalytic system also required further optimization. As shown in Additional file 1: Fig. S3, 35 mM ADHP and pH 7.0 were chosen as the optimal catalytic conditions. The detection sensitivity of CEAS fluorescence detection system was investigated under the optimum conditions. These results revealed that the fluorescence signal maintained a favorable linear relationship with the series concentration values in the 8 orders of magnitude HPV concentration range of 1 fM–10 nM, and the fitted equation was Y = 58.57 × (lg *c*) + 147.78 (R^2^ = 0.9928), where the limit of detection was calculated as 0.749 fM (Fig. 4D, E).

Similarly, to further validate the analytical performance parameters of the CEAS fluorescence detection system, the detection specificity and stability were explored. The cyclic recognition of identification probe allowed this strategy to avoid the interference of homologous viral nucleic acids (Fig. 4F). In addition, in contrast to the harsh reaction conditions and inactivation of proteases, the CEAS catalytic probe could maintain long-term stability, making the system signal stable at 7 days (Fig. 4G). Finally, standard spike-recovery experiments were performed in clinical serum samples to verify the system's ability to resist interference from complex matrices, and the results showed spike-recovery rates of 98.1–103.10% with RSD of 1.45–3.06% (Additional file 1: Table S3), indicating the potential clinical application of this fluorescent assay system.

## Conclusion

In summary, this study constructs a novel Controllable Enzyme Activity Switch, CEAS, by effectively regulating the aggregation state of the catalytic activity center hemin, and applies it to multimodal biosensing assays. The dimerization inactivation of hemin was ingeniously exploited to guide the tight binding of G4-Hemin DNAzyme and DNA-Hemin through DNA template in an orderly manner, thus constructing a target recognition-responsive regulatory structure for enzyme activity. G4-Hemin DNAzyme and DNA-Hemin synergistically exhibit high signal output capacity and are suitable for multimodal detection platforms. Moreover, a series of CEAS-based protein visualization assays or highly sensitive fluorescence assays, combined with aptamer recognition, RCA and SDR techniques have demonstrated excellent analytical performance. Compared with traditional protease assay technology, CEAS directly achieves the targeted activation and signal output of protein markers, shortens the detection time to 30 min, and greatly simplifies the detection process with a one-step operation. What’s more, CEAS allows highly sensitive detection of low-abundance nucleic acids with a minimum detection limit of 0.749 fM after coupling nucleic acid amplification technology, and also requires only 1 h reaction time (Additional file 1: Table S4). CEAS probe covers the whole process from target identification to signal output, which possesses the advantages of high sensitivity, rapidity, simplicity and low cost, and thus is expected to promote the innovation and upgrade of clinical enzyme assay system.

## Supplementary Information

**Additional file 1: Table S1.** Oligonucleotide sequences employed in this work. **Table S2.** The recoveries of thrombin using the proposed CEAS colorimetric catalytic system. **Table S3.** The recoveries of HPV using the proposed CEAS fluorescence catalytic system measured. **Table S4.** Comparison with recent methods for protein and nucleic acid based on different nanoenzyme or protease tag. Experimental section. **Figure S1.** The mass spectrum of DNA-Hemin sequences. **Figure S2.** PAGE electrophoresis validation of RCA amplification. **Figure S3.** Optimization of ADHP concentration A) and pH B) in CEAS fluorescence-catalyzed systems.

## Data Availability

All data generated or analyzed during this study are included in this article and the Additional Information. The additional file is available.

## References

[1] Shao Y, Zhou H, Wu Q, Xiong Y, Wang J, Ding Y (2021). Recent advances in enzyme-enhanced immunosensors. Biotechnol Adv.

[2] Gao L, Yang Q, Wu P, Li F (2020). Recent advances in nanomaterial-enhanced enzyme-linked immunosorbent assays. Analyst.

[3] Yoon BK, Sut TN, Yoo KY, Lee SH, Hwang Y, Jackman JA (2021). Lipid bilayer coatings for rapid enzyme-linked immunosorbent assay. Appl Mater Today.

[4] Cheng CM, Martinez AW, Gong J, Mace CR, Phillips ST, Carrilho E, Mirica KA, Whitesides GM (2010). Paper-based ELISA. Angew Chem Int Ed Engl.

[5] Gu K, Song Z, Zhou C, Ma P, Li C, Lu Q (2022). Development of nanobody-horseradish peroxidase-based sandwich ELISA to detect Salmonella Enteritidis in milk and in vivo colonization in chicken. J Nanobiotechnol.

[6] Kim Y-J, Choi J-W (2022). Enzyme-linked aptamer-based sandwich assay (ELASA) for detecting *Plasmodium falciparum* lactate dehydrogenase, a malarial biomarker. RSC Adv.

[7] Li Y, Sun L, Zhao Q (2019). Aptamer-structure switch coupled with horseradish peroxidase labeling on a microplate for the sensitive detection of small molecules. Anal Chem.

[8] Wei H, Wang E (2013). Nanomaterials with enzyme-like characteristics (nanozymes): next-generation artificial enzymes. Chem Soc Rev.

[9] Lin Y, Ren J, Qu X (2014). Catalytically active nanomaterials: a promising candidate for artificial enzymes. Acc Chem Res.

[10] Wu J, Wang X, Wang Q, Lou Z, Li S, Zhu Y (2019). Nanomaterials with enzyme-like characteristics (nanozymes): next-generation artificial enzymes (II). Chem Soc Rev.

[11] Ren X, Chen D, Wang Y, Li H, Zhang Y, Chen H (2022). Nanozymes-recent development and biomedical applications. J Nanobiotechnol.

[12] Gao L, Zhuang J, Nie L, Zhang J, Zhang Y, Gu N (2007). Intrinsic peroxidase-like activity of ferromagnetic nanoparticles. Nat Nanotechnol.

[13] Zhao W, Zhang R, Xu S, Cai J, Zhu X, Zhu Y (2018). Molecularly imprinted polymeric nanoparticles decorated with Au NPs for highly sensitive and selective glucose detection. Biosens Bioelectron.

[14] Zhu J, Du H-F, Zhang Q, Zhao J, Weng G-J, Li J-J, Zhao J-W (2019). SERS detection of glucose using graphene-oxide-wrapped gold nanobones with silver coating. J Mater Chem C.

[15] Cozma I, McConnell EM, Brennan JD, Li Y (2021). DNAzymes as key components of biosensing systems for the detection of biological targets. Biosens Bioelectron.

[16] Wang X, Qin L, Lin M, Xing H, Wei H (2019). Fluorescent graphitic carbon nitride-based nanozymes with peroxidase-like activities for ratiometric biosensing. Anal Chem.

[17] Zhu M, Chai Y, Yuan R, Zu B, Yuan Y (2021). Dual catalytic hairpin assembly and enzyme cascade catalysis amplification based sensitive dual-mode biosensor with significantly enhanced opposite signal readout. Sens Actuators B Chem.

[18] Wang F, Lu C-H, Willner I (2014). From cascaded catalytic nucleic acids to enzyme–DNA nanostructures: controlling reactivity, sensing, logic operations, and assembly of complex structures. Chem Rev.

[19] Kuzyk A, Schreiber R, Fan Z, Pardatscher G, Roller E-M, Högele A (2012). DNA-based self-assembly of chiral plasmonic nanostructures with tailored optical response. Nature.

[20] Cheng E, Xing Y, Chen P, Yang Y, Sun Y, Zhou D (2009). A pH-triggered, fast-responding DNA hydrogel. Angew Chem Int Ed.

[21] Lee H, Lytton-Jean AKR, Chen Y, Love KT, Park AI, Karagiannis ED (2012). Molecularly self-assembled nucleic acid nanoparticles for targeted in vivo siRNA delivery. Nat Nanotechnol.

[22] Fu J, Liu M, Liu Y, Woodbury NW, Yan H (2012). Interenzyme substrate diffusion for an enzyme cascade organized on spatially addressable DNA nanostructures. J Am Chem Soc.

[23] Liu Y, Du J, Yan M, Lau MY, Hu J, Han H (2013). Biomimetic enzyme nanocomplexes and their use as antidotes and preventive measures for alcohol intoxication. Nat Nanotechnol.

[24] Zhao Z, Fu J, Dhakal S, Johnson-Buck A, Liu M, Zhang T (2016). Nanocaged enzymes with enhanced catalytic activity and increased stability against protease digestion. Nat Commun.

[25] Liu M, Fu J, Hejesen C, Yang Y, Woodbury NW, Gothelf K (2013). A DNA tweezer-actuated enzyme nanoreactor. Nat Commun.

[26] Chen J, Cheng M, Salgado GF, Stadlbauer P, Zhang X, Amrane S (2021). The beginning and the end: flanking nucleotides induce a parallel G-quadruplex topology. Nucleic Acids Res.

[27] Ravichandran S, Razzaq M, Parveen N, Ghosh A, Kim KK (2021). The effect of hairpin loop on the structure and gene expression activity of the long-loop G-quadruplex. Nucleic Acids Res.

[28] Zhang K, Lv S, Lin Z, Li M, Tang D (2018). Bio-bar-code-based photoelectrochemical immunoassay for sensitive detection of prostate-specific antigen using rolling circle amplification and enzymatic biocatalytic precipitation. Biosens Bioelectron.

[29] Li J, Wu H, Yan Y, Yuan T, Shu Y, Gao X (2021). Zippered G-quadruplex/hemin DNAzyme: exceptional catalyst for universal bioanalytical applications. Nucleic Acids Res.

[30] Wang Q, Xu N, Lei J, Ju H (2014). Regulative peroxidase activity of DNA-linked hemin by graphene oxide for fluorescence DNA sensing. Chem Commun.

[31] Wang Q, Xu N, Gui Z, Lei J, Ju H, Yan F (2014). Catalytic activity of a dual-hemin labelled oligonucleotide: conformational dependence and fluorescent DNA sensing. Chem Commun.

[32] Cai Y, Li N, Kong D-M, Shen H-X (2013). Fluorogenic substrate screening for G-quadruplex DNAzyme-based sensors. Biosens Bioelectron.

[33] Serec K, Babić SD, Podgornik R, Tomić S (2016). Effect of magnesium ions on the structure of DNA thin films: an infrared spectroscopy study. Nucleic Acids Res.

[34] Deb A, Nalkar GR, Chowdhury D (2023). Biogenic carbon dot-based fluorescence-mediated immunosensor for the detection of disease biomarker. Anal Chim Acta.

[35] Gong W, Yang S, Zhang F, Tian F, Chen J, Yin Z (2021). A dual-quenched ECL immunosensor for ultrasensitive detection of retinol binding protein 4 based on luminol@AuPt/ZIF-67 and MnO_2_@CNTs. J Nanobiotechnol.

[36] Fonollosa J, Vergara A, Huerta R, Marco S (2014). Estimation of the limit of detection using information theory measures. Anal Chim Acta.

