# Supplementary material for: Target response controlled enzyme activity switch for multimodal biosensing detection
Source: J Nanobiotechnology. 2023 Apr 8;21:122. doi: 10.1186/s12951-023-01860-z (PMC10082497; doi:10.1186/s12951-023-01860-z)
Supplement: Supplementary file 1 — Additional file 1: Table S1. Oligonucleotide sequences employed in this work. Table S2. The recoveries of thrombin using the proposed CEAS colorimetric catalytic system. Table S3. The recoveries of HPV using the proposed CEAS fluorescence catalytic system measured. Table S4. Comparison with recent methods for protein and nucleic acid based on different nanoenzyme or protease tag. Experimental section. Figure S1. The mass spectrum of DNA-Hemin sequences. Figure S2. PAGE electrophoresis validation of RCA amplification. Figure S3. Optimization of ADHP concentration A) and pH B) in CEAS fluorescence-catalyzed systems. [file 12951_2023_1860_MOESM1_ESM.docx]

**Additional file 1**

**Target Response Controlled Enzyme Activity Switch for Multimodal Biosensing Detection**

Lu Zhang^a,1^, Haiping Wu ^a,1^, Yirong Chen^a^, Songzhi Zhang^a^, Mingxuan Song^a^, Changjin Liu^b^, Jia Li^c,^*, Wei Cheng^c,^*, Shijia Ding ^a,^*.

^a^ *Key* *Laboratory of Clinical Laboratory Diagnostics (Ministry of Education), College of Laboratory Medicine, Chongqing Medical University, Chongqing 400016, P.R. China*

^b^ *Department of Laboratory Medicine, The Fifth People's Hospital of Chongqing, Chongqing, 400062, China*

^c^ *The Center for Clinical Molecular Medical detection, The First Affiliated Hospital of Chongqing Medical University, Chongqing 400016, P.R. China*

*Correspondence: [dingshijia@163.com](mailto:dingshijia@163.com) (Shijia Ding), [chengwei@hospital.cqmu. edu.cn](mailto:chengwei@hospital.cqmu.%20edu.cn) (Wei Cheng) and [lijia20210203@163.com(Jia](mailto:lijia20210203@163.com(Jia) Li)

Tel: +86-23-68485688; Fax: +86-23-68485786.

^1^ Lu Zhang and Haiping Wu contributed equally to this work.

**Table S1. The synthetic DNA oligonucleotide sequences used in this method**

| Name | Sequence (5’-3’) |
| --- | --- |
| DNA-Hemin (0 base) | GGTGTCGCGTCCCTCGGCGGAA-Hemin^a^ |
| DNA-Hemin (1 base) | GGTGTCGCGTCCCTCGGCGGA-Hemin^a^ |
| DNA-Hemin (2 base) | GGTGTCGCGTCCCTCGGCGG-Hemin^a^ |
| DNA-Hemin (-1 base) | GGTGTCGCGTCCCTCGGCGGAAT-Hemin^a^ |
| DNA-Hemin (-2 base) | GGTGTCGCGTCCCTCGGCGGAATT-Hemin^a^ |
| G4-Hemin (B chain) | Hemin^a^-AAGGGTGGGTGGGTGGGTTTGGATGCCAC CCACCCTTTTCCGCCG |
| Guidance template | AGGGACGCCGACACCAGGACACCA |
| Guidance  template-aptamer | AGGGACGCCGACACCAGGACACCAGGTTGGTGTGGTTGG |
| primer | GTCGCGTCCCTCGGCGGAA |
| Padlock probe | Phos^b^-GGACGCGACACCAGGACACCATAATGCTATTTTTCCACCCACCCTTCCGCCGAG |

**Note:** a: Hemin-modified functional DNA sequences; b: 5' phosphate modified DNA sequence

**Table S2. The recoveries of thrombin using the proposed CEAS colorimetric catalytic system**

| **Sample** | **Addition**  **[pM]** | **Absorbance**  **[a.u.]** | **Found**  **[pM]** | **RSD**  **[%, n=3]** | **Recovery**  **[%, n=3]** |
| --- | --- | --- | --- | --- | --- |
| **1** | 10^4^ | 0.6405 | 10324.285 | 2.45 | 103.2 |
| **2** | 10^1^ | 0.3347 | 10.1827 | 1.29 | 101.8 |
| **3** | 10^-1^ | 0.13012 | 0.0993 | 1.78 | 99.3 |

**Table S3. The recoveries of HPV using the proposed CEAS fluorescence catalytic system measured**

| **Sample** | **Addition**  **[fM]** | **F.L**  **[a.u.]** | **Found**  **[fM]** | **RSD**  **[%, n=3]** | **Recovery**  **[%, n=3]** |
| --- | --- | --- | --- | --- | --- |
| **1** | 10^7^ | 558.476 | 10291932.94 | 1.45 | 102.9 |
| **2** | 10^3^ | 381.556 | 9809.5045 | 2.19 | 98.1 |
| **3** | 10^-1^ | 208.476 | 10.31 | 3.06 | 103.1 |

**Table S4. Comparison with recent methods for protein and nucleic acid based on different nanoenzyme or protease tag.**

| Enzymes type | Analyte | Platform | Detection limit | Detection time | Ref. |
| --- | --- | --- | --- | --- | --- |
| Nanozymes (MOF-818) | Thrombin | Colorimetric | 6.4pM | 1h | [1] |
| DNAzyme | Thrombin | Fluorescence | 1pM | 6.5h | [2] |
| Nanozymes (AuNPs) | Thrombin | Colorimetric | 0.9nM | 30min | [3] |
| DNAzyme(G4/Hemin) | Thrombin | Colorimetric | 4pM | 50min | [4] |
| DNAzyme(G4/Hemin) | HPV | Fluorescence | 2.3 pM | 3h | [5] |
| Nanozymes (AgNPs) | HPV | Colorimetric | 1.03nM | - | [6] |
| Protease (Endonuclease) | HIV | Colorimetric | 1.22nM | 3.5h | [7] |
| This work | Thrombin  HPV | Colorimetric  Fluorescence | 1.8pM  0.749fM | **30min**  **60min** |  |

**Experimental section**

**Catalytic Rate Measurements and Calculation.**

The peroxidase activity of G4/hemin DNAzymes was measured by monitoring the absorbance change at 585 nm for ADHP, which reflect the oxidation rate of ADHP by H2O2 accompanied by the generation of fluorescent oxidized product high fluorescent resorufin. Absorbance versus time profiles were obtained by using a UV-2550 spectrophotometer (Shimadzu, Japan) for 300s in 10mm quartz cuvette. The G4-hemin complexes were prepared by incubating various preformed G4s or CEAS (100nM of each group) in Tris-HCl buffer for 1 h at 37 °C, and reactions were initiated by the addition of 4mM TMB or 35mM ADHP and 50 mM H2O2. The initial rates (V0) were obtained based on the slopes of the initial linear portion (the first 400 s or 60s) of the increase in the absorbance and the ε value (about 39 000 M ^-1^∙cm^-1^ for TMB at 650 nm and 54 000 M ^-1^∙cm^-1^ for ADHP at 585nm). All kinetic measurements were measured at least three times.

**Specificity test of the CEAS for target detection.**

The specificity of the proposed CEAS-based visualization assay system for protein biomarker detection was evaluated to demonstrate its practicability. For this purpose, various substances such as Lzm (Lysozyme), GOx (Glucose oxidase) and BSA (Bovine serum albumin) were selected as potential interferences. The concentration of thrombin was chosen as 1 ng mL^−1^, and all interfering substances as 1ng mL^−1^.

**Stability of the CEAS for target detection.**

For the stability test, whether protein or nucleic acid target, after termination of the SDR reaction, the 100 nm CEAS was depolymerized into G4-Hemin and DNA-Hemin and stored in the dark at 4 °C. Absorbance and fluorescence values were measured for 7 consecutive days to obtain assay stability. Each sample was repeated three times.


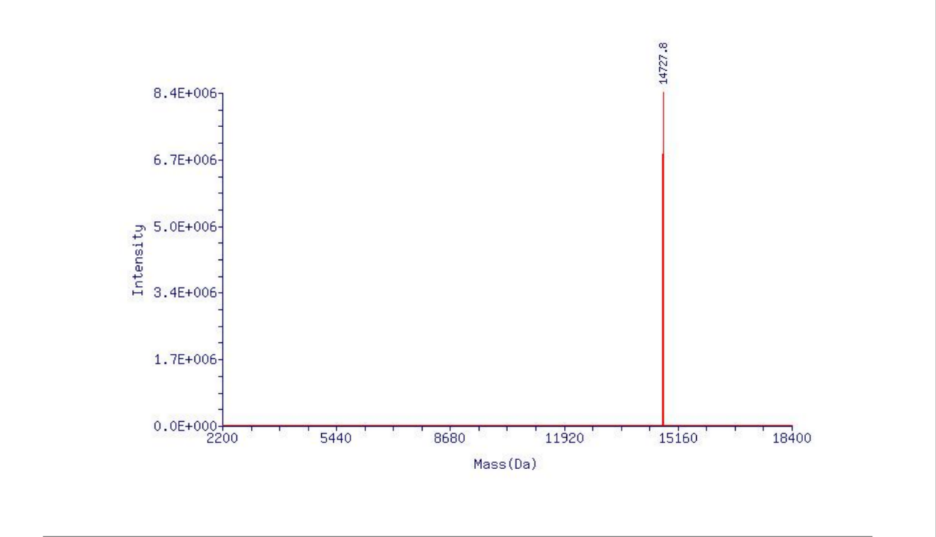


**Fig. S1.** The mass spectrum of DNA-Hemin sequences.


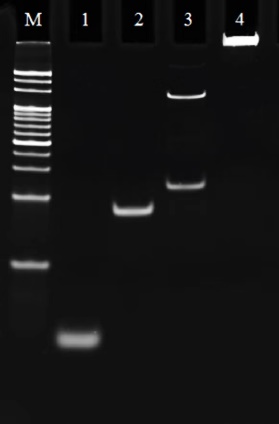


**Fig. S2** PAGE electrophoresis validation of RCA amplification. Lane 1: primer; Lane 2: padlock probe; Lane 3: cyclization product; Lane 4: RCA products. The band marked by the red box was the RCA products, because the number of bases in the products was too long, so the band had a significant back shift compared to the linear template. Therefore, the presence of RCA products in lane 4 indicated that the synthesis of RCA was successful. Of the two bright bands appearing in lane 3, the lower band was a combination of 1 and 2, while the macromolecular band above was probably due to dimer production.


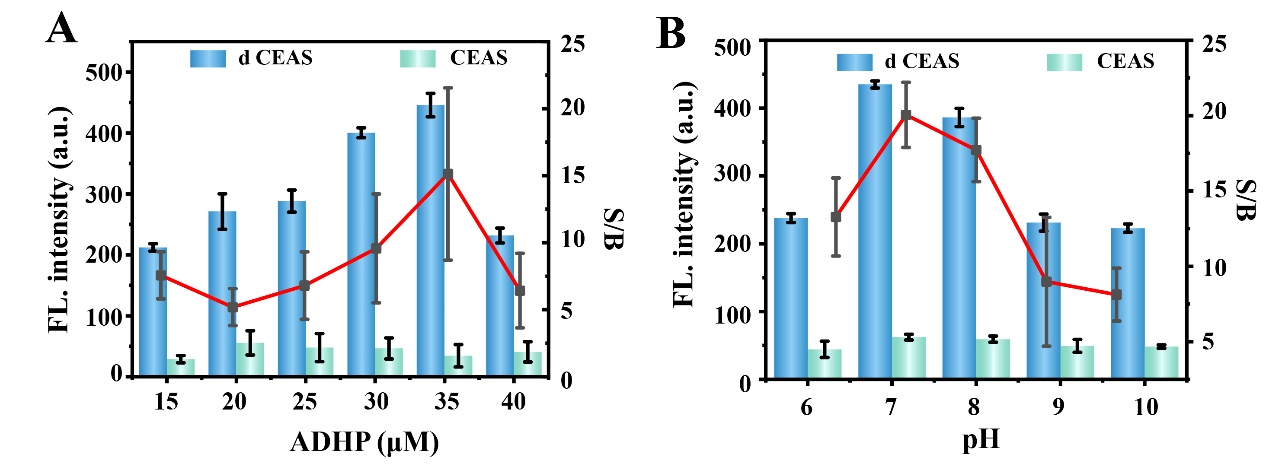


**Fig. S3** Optimization of ADHP concentration A) and pH B) in CEAS fluorescence-catalyzed systems.

**References**

1. Jiang J, Wang Y, Kan X. A facile nanozyme based catalytic platform for the selective and sensitive detection of thrombin. Microchemical Journal. 2022;172:106965.

2. Yun W, Li N, Wang R, Yang L, Chen L, Tang Y. Proximity ligation assay induced hairpin to DNAzyme structure switching for entropy-driven amplified detection of thrombin. Analytica Chimica Acta. 2019;1064:104–11.

3. Wu T, Li X-Y. An instrument-free visual quantitative detection method based on clock reaction: the detection of thrombin as an example. Anal Methods. 2023;15:48–55.

4. Liu M, Li J, Li B. A colorimetric aptamer biosensor based on cationic polythiophene derivative as peroxidase mimetics for the ultrasensitive detection of thrombin. Talanta. 2017;175:224–8.

5. Chen J, Wang M, Zhou C, Zhang J, Su X. Label-free and dual-mode biosensor for HPV DNA based on DNA/silver nanoclusters and G-quadruplex/hemin DNAzyme. Talanta. 2022;247:123554.

6. Teengam P, Siangproh W, Tuantranont A, Vilaivan T, Chailapakul O, Henry CS. Multiplex Paper-Based Colorimetric DNA Sensor Using Pyrrolidinyl Peptide Nucleic Acid-Induced AgNPs Aggregation for Detecting MERS-CoV, MTB, and HPV Oligonucleotides. Anal Chem. 2017;89:5428–35.

7. Wang X, Huang Z, Chen J, Luo Z, Xu Y, Duan Y. A colorimetric sensing platform based on site-specific endonuclease IV-aided signal amplification for the detection of DNA related to the human immunodeficiency virus. Anal Methods. 2019;11:2190–6.
